# Nano-Floating Gate Memory Devices Composed of ZnO Thin-Film Transistors on Flexible Plastics

**DOI:** 10.1007/s11671-010-9789-5

**Published:** 2010-09-28

**Authors:** Byoungjun Park, Kyoungah Cho, Sungsu Kim, Sangsig Kim

**Affiliations:** 1 Department of Electrical Engineering, Korea University, 5-1, Anam-dong, Sungbuk-gu, Seoul 136-701, Korea

**Keywords:** Nanoparticles, Thin-film transistors, Low temperature, Non-volatile memory, Flexible devices

## Abstract

Nano-floating gate memory devices were fabricated on a flexible plastic substrate by a low-temperature fabrication process. The memory characteristics of ZnO-based thin-film transistors with Al nanoparticles embedded in the gate oxides were investigated in this study. Their electron mobility was found to be 0.18 cm^2^/V·s and their on/off ratio was in the range of 10^4^–10^5^. The threshold voltages of the programmed and erased states were negligibly changed up to 10^3^ cycles. The flexibility, memory properties, and low-temperature fabrication of the nano-floating gate memory devices described herein suggest that they have potential applications for future flexible integrated electronics.

## Introduction

Flexible electronic devices have attracted considerable attention because they enable remarkable applications such as flexible display backplanes, electronics papers, and radio frequency identification tags, due to their light weight, low cost, and low profile [1,2]. Currently, amorphous silicon (a-Si), polycrystalline silicon and organic semiconductors are the dominant materials used for transistors in these systems [3,4]. However, an emerging trend in flexible electronics is the development of alternative materials with the goal of increasing their mobilities, decreasing their cost, ensuring their chemical stability, and enabling them to be fabricated on low-cost, flexible substrates with a low-temperature process.

To realize high carrier mobilities, excellent chemical stability, and a low-temperature process, many inorganic semiconductors have been researched [5,6]. Especially, oxides of transition metals including ZnO and IGZO provide the possibility for transparent and flexible electronics to be developed [7,8]. These oxides are usually transparent in the visible region, owing to their large band-gaps (over 3 eV), and their optical properties provide for outstanding applications, such as invisible electronics for transparent displays on automobile windshields, when the oxide films are combined with transparent conducting oxides and insulators. Such oxide materials with properties suitable for thin-film transistors (TFTs) can be formed on flexible plastic substrates by the rf-sputtering method, pulsed-laser deposition, and solution processes at low temperature [9-11]. The mobilities that can be achieved using the above-mentioned processes are in the range of 11–80 cm^2^/V·s [12,13]. However, the focus of recently reported studies has been on enhancing the TFT performance, such as the carrier mobility and on/off ratio. It is useful to investigate other circuit components, such as transparent diodes and memory elements, to extend the concept of transparent or flexible transistors to circuits and electronics.

In this paper, we demonstrate the fabrication processes and electrical characteristics of flexible nonvolatile memory devices based on bottom-gate ZnO TFTs consisting of transparent floating gate transistors that incorporate Al NPs which act as the floating gate layers in the gate dielectric. The bottom-gate nano floating gate memory (NFGM) devices could be fabricated on any plastic substrates so that the application area of NFGM could be expanded to wearable electronic devices.

## Experimental

Bottom-gate TFTs with 60-nm-thick ZnO channels and 50-nm-thick SiO_2_ gate insulators embedded with Al NPs were fabricated on PES (polyether sulfone) plastic substrates at room temperature. The SiO_2_ layers were deposited on the unheated plastic substrates at an rf-power of 100 W, gas mixing ratio of Ar/O_2_ of 4/5, and total pressure of 3.5 mTorr. The sputtering durations were 6 min for the ZnO and 60 min for the SiO_2_ layers. The TFT structure was defined using photolithography and lift-off processes. The electrodes were formed with sputtered Al layers. High-resolution transmission electron microscopy (HRTEM) and energy dispersive X-ray (EDX) spectroscopy were used to analyze the cross-section of the film. The output and transfer characteristics of the fabricated ZnO/Al-NPs memory TFTs were measured with a semiconductor parameter analyzer (Agilent 4155C) and a pulse generator (Agilent 41501B) and the bending properties of the flexible memory device were also measured. The bending tests were performed using a home-made bending machine, and the memory characteristics were measured after the devices suffered from the bending cycles consisting of tensile strained and unstrained states.

## Results and Discussion

**Figure 1:**
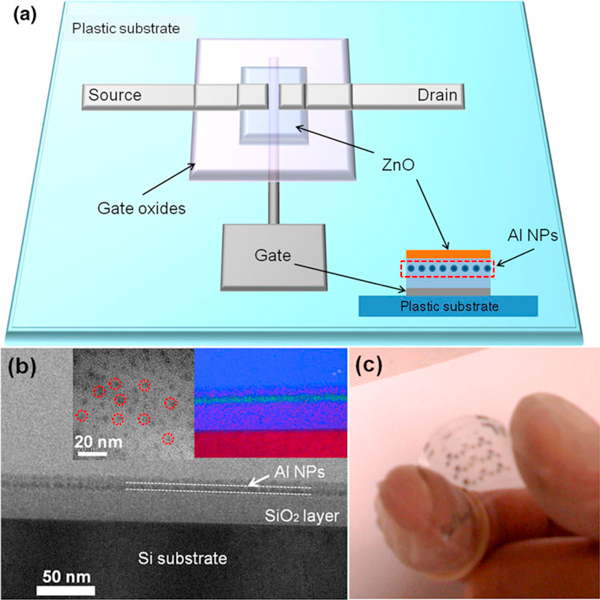
**A schematic diagram of the flexible ZnO/Al-NPs memory TFT and the cross-sectional device structure (*inset*) (a), and a cross-sectional TEM image of the stacked gate layer (b)**. The *left inset* is a planar HRTEM image of Al NPs on the SiO_2_ layer, and the right inset shows the EDX elemental mapping of Al (*cyan*), Si (*red*), and O (*blue*). A photographic image showing the ZnO/Al-NPs memory TFTs on a flexible plastic substrate (**c**).

A schematic diagram of a representative bottom-gate ZnO TFT with Al NPs embedded in gate oxides (or a ZnO/Al-NPs memory TFT) is shown in Figure 1a. A cross-sectional HRTEM image of the stacked gate layer is exhibited in Figure 1b. The inset in Figure 1b shows the corresponding EDX elemental mapping from the HRTEM image. The EDX elemental mapping for the stacked film with a thickness of 50 nm reveals the presence of Al (cyan), Si (red), and O (blue) atoms, which originate from the embedded Al NPs between the SiO_2_ layers. Figure 1c depicts a photographic image of the ZnO/Al-NPs memory TFTs on a flexible plastic sheet.

**Figure 2:**
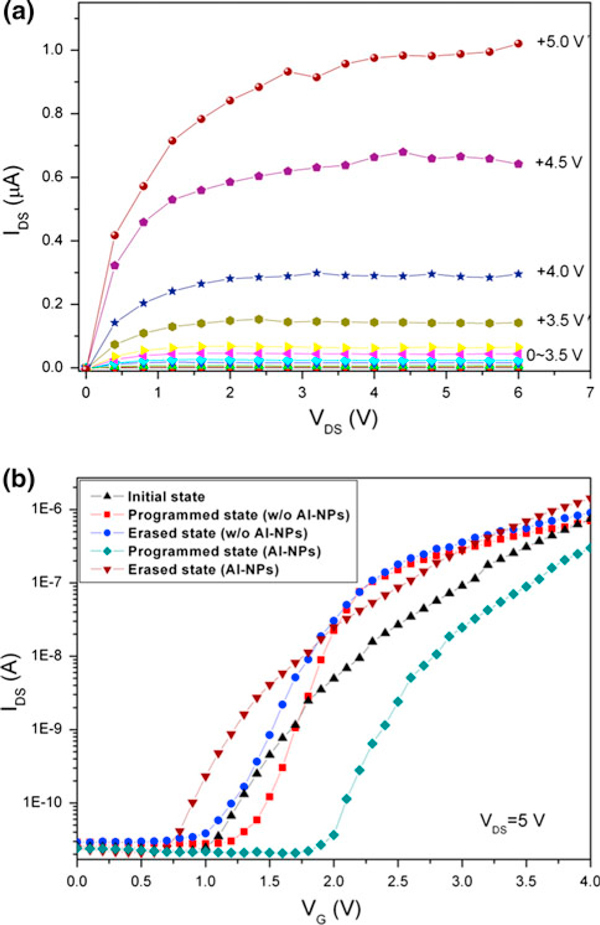
**a Output characteristics of the flexible ZnO/Al-NPs memory TFT at selected gate voltages, and b transfer characteristics of the flexible ZnO/Al-NPs memory TFT and the reference sample (without any Al NPs)**.

The output characteristics of a representative ZnO/Al-NPs memory TFT are shown in Figure 2a. This device operates in enhancement mode as an n-type field-effect transistor showing good performance and the on/off ratio of its drain-to-source current is 10^4^ with the off current being as low as 10^-10^ A. The ZnO/Al-NPs memory TFT exhibits a sub-threshold gate voltage swing of 0.5 V/decade and the saturation mobility is estimated to be 0.18 cm^2^/V·s. The transfer characteristics of the unit cell are also exhibited in Figure 2b. A threshold voltage shift of 1.2 V is estimated for the ZnO/Al-NPs memory TFT when the gate voltage is swept from 0 V to a positive voltage of 5 V, whereas the transfer property of the reference sample (without any Al NPs) shows a threshold voltage shift of 0.3 V. The shift of the ZnO/Al-NPs memory TFT is due to the charge trapping in the NPs through the tunneling oxide layer. The threshold voltage shift of the reference sample comes from the charge trapping sites existing in the interface between the channel layer and control oxide layer or in the sputtered SiO_2_ layers.

**Figure 3:**
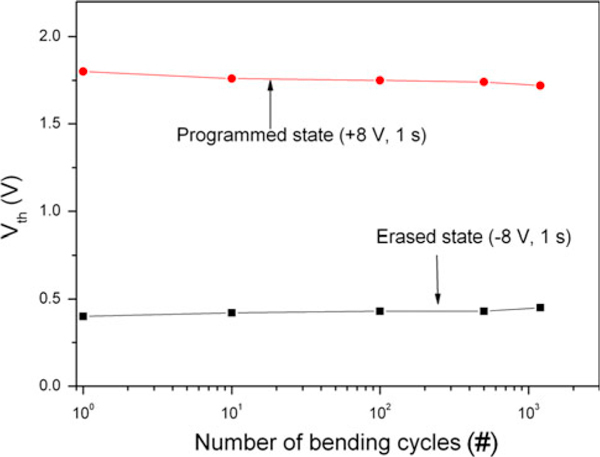
**Threshold voltages in programmed/erased states as a function of the number of bending cycles**.

In order to examine the effect of bending on the threshold voltage shift of the flexible ZnO/Al-NPs memory TFT, the magnitudes of the programmed and erased states were measured as shown in Figure 3. Considering breakdown of the sputtered SiO_2_ layer used as the tunneling oxide in this study, the device was programmed and erased by applying the voltage of ±8 V for a pulse width of 1 s. The quality of the sputtered SiO_2_ layer without any post-annealing is poorer than that made from thermal oxidation; note that the sputtering method without any post-annealing is utilized in this work for the fabrication of the devices on temperature-sensitive substrates. The magnitude of the tensile strain stress on the bent substrate is 0.5% and the strain is estimated using the following equation;

(1) $$\frac{\left(thickness\;of\;the\;substrate+total\;thickness\;of\;films\right)}{2\times R_{c}}\times 100$$

where R_c_ corresponds to the bending curvature radius of the substrate and the thickness of the substrate and total thickness of the film are 200 μm and 240 nm, respectively [14]. Here, the total thickness of the film corresponds to the thickness of the device including the channel layer, electrodes and the gate oxide layers. The substrate experiences a strain of 0.5% when R_c_ is 19.5 mm. In this measurement, the substrate is bent from a flat condition to a tensile strain of 0.5%. The endurance of the flexible ZnO/Al-NPs memory TFT is investigated by means of the continuous substrate bending test. The ratio of the two states, the programmed and erased states, is negligibly changed up to one thousand cycles.

**Figure 4:**
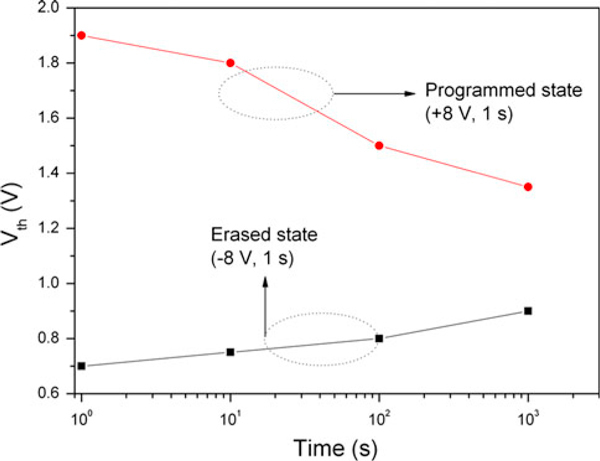
**Retention characteristics of the ZnO/Al-NPs memory TFT on the plastic substrate**.

The retention properties of the flexible ZnO/Al-NPs memory TFT are demonstrated in Figure 4. The charge retention characteristics were measured at room temperature after stressing the flexible ZnO/Al-NPs memory TFT with a gate voltage of +8 V for 1 s, while the source and drain electrodes were grounded. The retention characteristics were also measured after erasing the device with a gate voltage of -8 V. After 10^3^ s, there was a threshold voltage shift of 0.35 V, which is insufficient for commercial nonvolatile memory devices. The poor retention characteristics are ascribed to the inferior quality of the SiO_2_ layers deposited by the sputtering method without any post-annealing. The charge loss from the floating gate layer through the gate oxide layers could be reduced by replacing the sputtered SiO_2_ layer with a polymer insulator; note that polymer insulating materials, such as parylene-C introduced in our previous work [15], will be utilized in future works to reduce the charge loss from the floating gate layer without the need for a thermal process. The flexible ZnO/Al-NPs memory TFT structure with a polymer insulating layer would exhibit better retention characteristics.

## Conclusion

NFGM devices were fabricated on a flexible plastic substrate for the first time. The process used for making the Al NPs-embedded TFTs on the gate oxides was performed at room temperature. Their electron mobility was found to be 0.18 cm^2^/V·s and their on/off ratio was of the order of 10^4^. The threshold voltages of the programmed and erased states were negligibly changed up to 10^3^ cycles. The techniques developed in this work demonstrate that flexible electronic devices, including TFTs, nonvolatile memory chips and others, can be fabricated on temperature sensitive substrates.
